# The Effects of the Location of Au Additives on Combustion-generated SnO_2_ Nanopowders for CO Gas Sensing

**DOI:** 10.3390/s100707002

**Published:** 2010-07-21

**Authors:** Smitesh D. Bakrania, Margaret S. Wooldridge

**Affiliations:** 1 Department of Mechanical Engineering, Rowan University / 201 Mullica Hill Road, Glassboro, NJ 08028, USA; 2 Department of Mechanical Engineering, University of Michigan / 2350 Hayward Street, Ann Arbor, MI 48109, USA; E-Mail: mswool@umich.edu

**Keywords:** SnO_2_, gas sensor, additives, gold, combustion synthesis

## Abstract

The current work presents the results of an experimental study of the effects of the location of gold additives on the performance of combustion-generated tin dioxide (SnO_2_) nanopowders in solid state gas sensors. The time response and sensor response to 500 ppm carbon monoxide is reported for a range of gold additive/SnO_2_ film architectures including the use of colloidal, sputtered, and combustion-generated Au additives. The opportunities afforded by combustion synthesis to affect the SnO_2_/additive morphology are demonstrated. The best sensor performance in terms of sensor response (*S*) and time response (*τ*) was observed when the Au additives were restricted to the outermost layer of the gas-sensing film. Further improvement was observed in the sensor response and time response when the Au additives were dispersed throughout the outermost layer of the film, where *S* = 11.3 and *τ* = 51 s, as opposed to Au localized at the surface, where *S* = 6.1 and *τ* = 60 s.

## 1. Introduction

Environmental monitoring is increasingly becoming the standard in the industrial, residential and commercial sectors; fueled by our growing awareness of gases or vapors that are harmful to human health or the environment. Solid state metal oxide gas sensors are ideally suited for such gas-sensing applications because of their compact size, ruggedness and low power consumption. Research on tin dioxide (SnO_2_), zinc oxide (ZnO), zirconia (ZrO_2_), and titania (TiO_2_) based gas sensors continues to introduce sensors with better sensor response, time response and selectivity by focusing on the film composition and architecture including characteristics such as trace additives or dopants, film morphology, and surface treatments [1–6]. Among the SnO_2_ additives considered, gold (Au) has been demonstrated to dramatically improve tin dioxide gas sensors in terms of sensor response and selectivity to some target gases [7–17].

The role of additives on the fundamental chemical and physical mechanisms important during gas sensing remains highly uncertain [17]. Changes in the electronic, chemical and physical properties of the SnO_2_ have been proposed ([3] and refs therein). The experimental and theoretical efforts are complicated by the issue that often only bulk loadings of the additives are reported, and studies have shown the location and the relative morphology of the materials can also have significant impact on sensor performance [18]. The objective of the current work was to systematically explore how controlling the distribution and location of gold nanoparticle additives can be used to alter and ultimately enhance tin dioxide gas sensor performance. Multiple integration methods are considered in this study to achieve a variety of film architectures.

## 2. Experimental

The Au nanoparticle additives considered in the study were integrated into the SnO_2_ sensors using multiple material synthesis and sensor film deposition procedures. All the SnO_2_ materials were generated using the combustion synthesis approached described previously [19]. Three methods were considered for generating the gold nanoparticles: combustion synthesis (CS), metal precipitation (MP) and sputtering (S). The following sections describe the materials synthesis and sensor fabrication methods used.

### 2.1. SnO_2_ Synthesis

**Figure 1.**
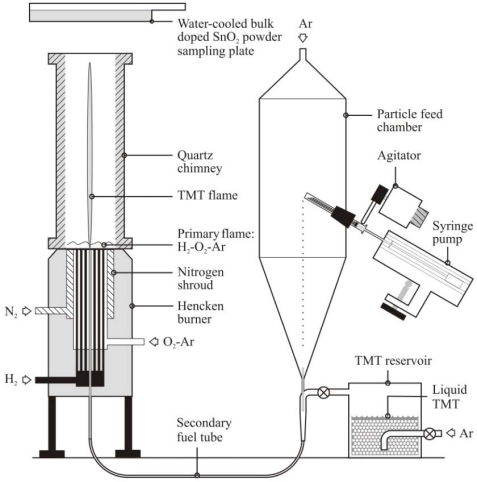
Schematic of the combustion synthesis (CS) facility used to generate the SnO_2_ powders and the CS generated Au additives.

The SnO_2_ sensing materials were fabricated using a combustion synthesis facility shown schematically in Figure 1 and described previously in Bakrania *et al.* [19–21] and Miller *et al.* [22]. Briefly, the SnO_2_ powders were generated using a hydrogen/oxygen/argon burner with reactant gas flow rates of 2.7/1.47/17.14 l pm. Liquid tetramethyl tin (TMT, (CH_3_)_4_Sn, 98% purity, Alfa Aesar) as the SnO_2_ precursor was injected into the hydrogen/oxygen/argon flame using a bubbler system. At the standard conditions used in the study (1 atm, 298 K), the TMT bubbler system yields an argon flow (63.5 mL/min) saturated with 21–23% TMT (mole basis). A water-cooled cold plate was used to collect the powders that were generated at a rate of approximately 1.6 g/h.

### 2.2. Combustion Synthesis of Au Additives

Using combustion synthesis methods, the additives can be simultaneously generated and integrated with the SnO_2_, as described in Bakrania *et al.* [20]. Briefly, the particle feed system shown in Figure 1 was used with gold acetate (99.9% purity, Alfa Aesar, sieved to <45 μm before use) to generate Au-doped SnO_2_. For these studies, the syringe pump was set to a constant injection rate of 1 mL/h of gold acetate. The gold acetate particles decompose rapidly in the H_2_/O_2_/Ar flame to form metallic gold nanoparticles [23]. A chimney was used to improve the capture efficiency of the Au-SnO_2_ powders produced by the particle feed system.

### 2.3. Metal Precipitation of Au

Colloidal gold was also used to dope the CS SnO_2_ powders. A colloidal suspension of gold was prepared from hydrogen tetrachloroaurate (HAuCl_4_, Sigma Aldrich) using the methods described by McFarland *et al.* [24]. The colloidal gold suspension was mixed with undoped CS SnO_2_ dispersion (described below) in a 1:10 volumetric ratio. Such a mixture produces approximately 0.2 wt.% gold, based on complete conversion of HAuCl_4_ to gold.

### 2.4. Sputtering of Au

Localizing the Au additive via sputtering (Denton Desk II) was investigated by depositing the Au onto the outermost surface of the SnO_2_ film. For these sensors, two layers of undoped CS SnO_2_ were first deposited onto the sensor platform (described in Section 2.5) and dried at ambient conditions. A 2 nm thick layer (based on instrument calibration) of Au was then deposited using a gold target with ionized argon. Following the sputtering step, the sensors were annealed in the furnace at 500 °C for 1.5 h.

### 2.5. Sensor Fabrication

**Figure 2.**
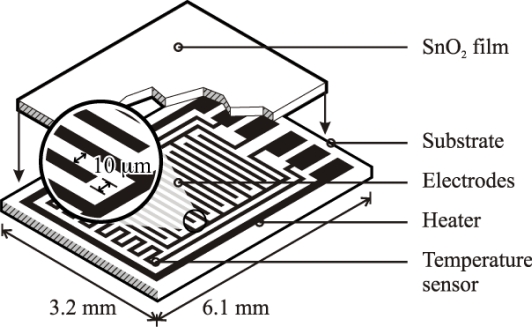
Schematic of the sensor platform (Heraeus MSP 632). The sensing and heating circuits are not drawn to scale.

Based on the high quality performance and the highly repeatable properties of the sensors, the novel dispersion-drop sensor fabrication process developed by Bakrania *et al.* [19] was used in this study. The binderless sensor fabrication process has been described previously [19]. A short summary is provided here. The sensing materials were deposited onto commercially available sensing platforms (Heraeus MSP 632), which were equipped with interdigitated platinum electrodes (10 μm electrode separation), heating circuits and temperature sensing circuits deposited on alumina substrates (see Figure 2). The calibration for the temperature sensing circuit was provided by the manufacturer. Each powder sample was ground using mortar and pestle prior to application to the sensing platform. The powders were then dispersed in an ethanol-water solution (15% C_2_H_5_OH in distilled water) using a sonic horn (Sonics VC-505 Ultrasonic processor) yielding ∼1.8 wt.% SnO_2_ in the dispersion. A micropipetter was used to deposit a single drop of 10 μL of the dispersion onto a clean sensor platform. The drop was allowed to evaporate at ambient conditions followed by a low-temperature heating step performed in a muffle furnace (Fisher Scientific) at 80 °C for half an hour. This was followed by another drop deposition step to add a second layer and another low-temperature heating step. Each sensor film consisted of two layers of tin dioxide, and each film was sintered at high temperature (500 °C for 1.5 h). Total film thickness, confirmed by SEM imaging, was 10 μm (with 5 μm per dispersion-drop layer).

### 2.6. Sensor Testing

Two digital flow meters (TSI 4100 Series) controlled the flow of gases into a mixing tank before flowing into a 600 mL glass chamber. The sensor tests were performed using dry air and a CO-dry air mixture (1,000 ppm CO, 99% purity, Cryogenic Gases) flowing at a total volumetric rate of 400 mL/min. All experiments were conducted by exposing the sensors to 500 ppm of CO in dry air. A DC power supply (BK Precision 1760A) was used to power the resistive heater. The temperature circuit resistance and the electrode resistance were measured using a Keithley 6487 Picoammeter/Voltage Source. Sensing measurements were performed after 24 h of conditioning each sensor at a fixed operating temperature of 330 ± 5 °C. The sensor response was defined as *S = R_a_/R_g_*, where *R_a_* is the resistance in air while *R_g_* is the resistance in target gas, or CO in this case. Time response (*τ*) was calculated using an algorithm that evaluated the time required for the sensor to achieve 90% of the final resistance value *R_g_* after CO exposure. The recovery time was similarly calculated and represented the time required for the sensor to achieve 90% of the end value after CO flow was stopped.

### 2.7. Materials Analysis

Samples were collected to characterize the materials properties of the doped SnO_2_ used in the sensors. Samples were simultaneously deposited onto glass slides to perform x-ray diffraction (XRD, Scintag Theta-Theta) analysis. Scans for phase identification and for average additive crystallite size were obtained using increments of 0.02° 2θ and CuKα radiation (λ = 1.5405 Å). The scans were obtained over a 2θ range of 20°–90° at a scan rate of 5° 2θ/min. Spectral scans for average crystallite size for SnO_2_ were measured over a 2θ range of 22°–48° at a scan rate of 0.5° 2θ/min. Peak positions and relative intensities of the powder patterns were identified by comparison with reference spectra [25]. The average crystallite size was determined from the XRD spectra using the Scherrer equation:

$$d_{\text{XRD}}=\frac{0.9\lambda }{\beta_{½}\text{cos}\;\theta }$$

where *d_XRD_* is the average crystallite size, *λ* is the source wavelength, *β_1/2_* is the full-width at half-maximum of the peak used for the analysis and *θ* is the XRD scattering angle of the peak.

Scanning electron microscopy (SEM, Philips XL30) and optical microscopy were used to characterize the film quality. SEM imaging was performed using either the samples deposited onto the glass slides or by direct imaging of the sensors. SEM was combined with x-ray energy dispersive spectroscopy (XEDS, SEM Philips XL30) for determining composition. Electron microprobe analysis (EMPA, Cameca SX100) was also used for compositional analysis of samples deposited onto glass slides.

Transmission electron microscopy (TEM, Philips CM-12) was used to determine nanoparticle morphology and size. TEM samples were prepared by depositing a drop of either the Au-doped SnO_2_ dispersions or the colloidal suspension onto TEM grids that were dried at ambient conditions. None of the TEM samples were sintered.

## 3. Results and Discussion

Four sensor architectures were considered with additives generated using combustion synthesis (CS), metal precipitation (MP) or sputtering (S) methods. These methods afforded two categories of additive distribution within the deposited film: additives distributed throughout the sensing film or additives localized away from the sensing electrodes. Table 1 provides a summary of the Au-doped sensors and the corresponding film characteristics. The sensors were each characterized in terms of the film nanoarchitecture (e.g., film composition, location of additives, *etc.*) and sensor performance (e.g., sensor response, time response and recovery time).

### 3.1. Characterization of Additives

**Figure 3.**
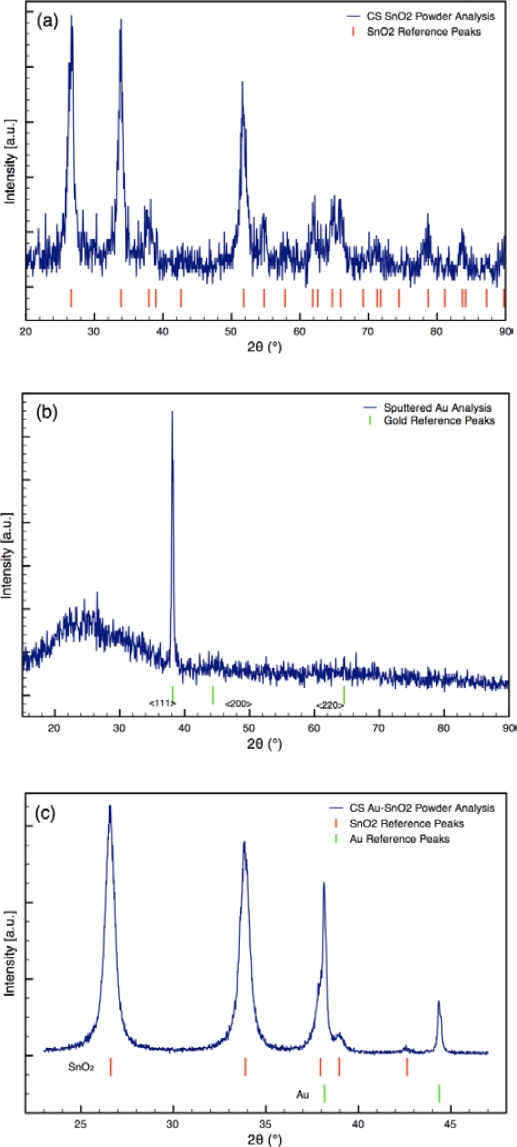
Comparison of typical XRD spectra of (a) undoped SnO_2_ film, (b) Sputtered (post heat treatment) Au film on glass substrate, Sensor C outermost layer and (c) CS Au-doped SnO_2_ film, Sensor A film & D outermost layer (typical high resolution XRD spectrum used for Scherrer analysis).

Typical XRD spectra of Au-doped CS SnO_2_ films are presented in Figure 3. The XRD analyses revealed metallic gold (4–784) and the cassiterite phase of tin dioxide (41–1445) for the sensor architectures [25]. The average crystallite sizes of the Au and SnO_2_ determined from the spectra are listed in Table 1. The size of the SnO_2_ particles was consistent for all sensor architectures. The size of the Au particles varied from 15 nm to 65 nm based on the synthesis process used.

The metal loadings were determined using SEM XEDS for the combustion-generated additives. SEM XEDS yielded elemental gold content of the Sensor A film (as-produced) of 3 wt.% in tin dioxide. The XEDS results were augmented by EMPA. The microprobe analysis was performed at approximately ten locations on a sample of the Sensor A film deposited on the glass slide with a probe sampling area of 20 μm × 30 μm. The EMPA results yielded mean loadings of 3.5 wt.% for the gold content in the sensing film, consistent with the SEM XEDS data on the as-produced film.

**Figure 4.**
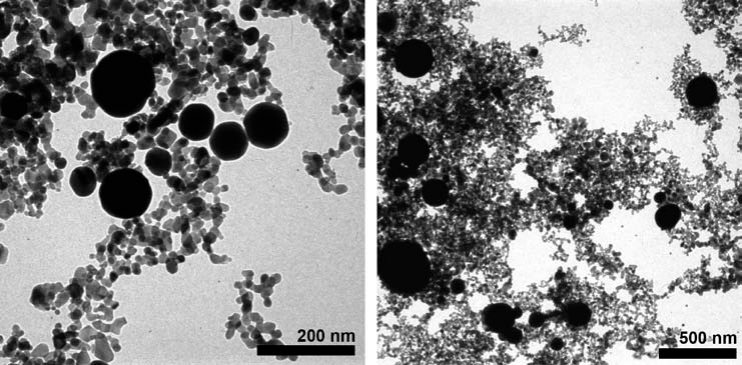
TEM images of gold-doped SnO_2_ film materials used in sensor architectures A and D. The large dark discrete spheres were identified as gold using XEDS.

TEM imaging was used to determine the morphology of the Au-SnO_2_ nanocomposites, including the distribution of the Au additives. Typical TEM images of the Au-SnO_2_ CS powders are presented in Figure 4. The gold particles observed using TEM were spherical and ranged from 50–300 nm in diameter in clusters of 10–30 discrete spherical particles at several locations in the sample. The TEM results suggest that even though the XEDS and EMPA results yielded *bulk* dopant loadings of 3.5 wt.% gold, the *local concentration* of gold nanoparticles can be substantially higher (e.g., 30–50 wt.% for an area with a diameter of 1 μm).

**Figure 5.**
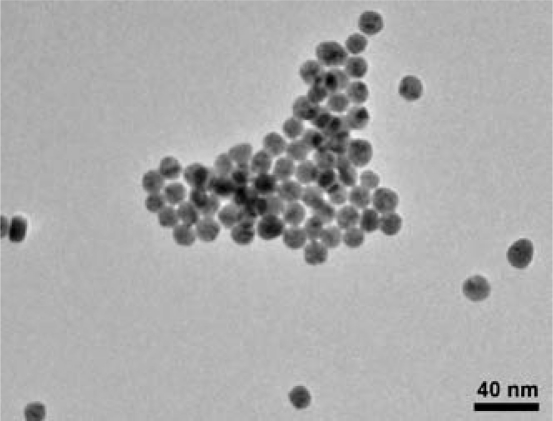
TEM image of colloidal gold nanoparticles used as additives in sensor architecture B.

TEM imaging of the Au particles made from the colloidal suspension is presented in Figure 5. As seen in the figure, the colloidal suspension yielded approximately 15 nm diameter spherical nanoparticles of gold with narrow size distribution. Figure 5 also shows that the gold nanoparticles clustered as they dried.

For the sensors (Sensor C) created using sputtering, the initial sputtering process results in a conformal layer of Au approximately 2 nm thick as the outermost layer of the sensor, prior to annealing. The outer layer of the film was golden in color before annealing; however, the surface was pink following annealing. The color change indicates the sputtered gold film coalesced into gold nanoparticles during annealing, which is consistent with the study by Mizsei *et al.* [26] who studied the agglomeration of metal particles that occurs during heat treating of sputtered metal layers deposited onto metal oxide surfaces. XRD analysis of the sputtered Au layer on glass slide confirms the gold layer forms nanoparticles during heat treatment (see Figure 3(b)). XRD Scherrer analysis results in an average Au particle size of 32 nm. Hence, the Au sputtering and heat treating processes yield sensors with gold nanoparticles localized at the outermost layer of the sensor.

### 3.2. Characterization of Sensor Performance: Distributed Au Additives

**Figure 6.**
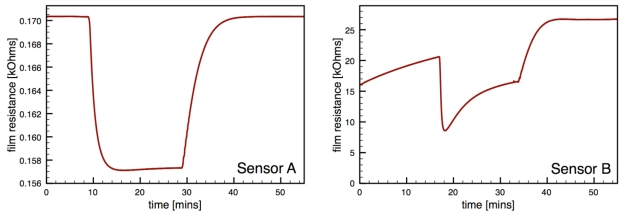
Sensor performance for distributed gold additives; sensor architectures A and B.

Fig. 6 presents typical sensor performance for the sensor architectures A and B with distributed Au additives. The CS Au-doped sensor has a baseline resistance of *R_a_* = 0.17 kΩ and a relatively low sensor response *S* = 1.08, compared to an equivalent undoped CS SnO_2_ sensor (*S* = 4.7) [19]. The Au sensor architecture A demonstrates well-behaved response with the film resistance returning to the baseline value after the CO flow is stopped. Sensor architecture B exhibits much higher baseline resistance and poor quality signal with drift in the baseline and hysteresis upon exposure to CO.

One possible explanation for the low film resistance is if the metal content of the SnO_2_ films (*i.e.,* the dopant loading) was sufficiently high, the metals could effectively act as a shunt, as suggested by other studies [27,28]. To test this hypothesis the dopant loading was reduced by dilution with an undoped SnO_2_ dispersion. The doped and undoped tin dioxide dispersions were mixed in a 1:10 volumetric ratio that leads to ten-fold reduction in dopant loading by weight. The diluted Au-doped SnO_2_ dispersion was deposited on sensing platforms for testing. The sensor response for each sensor remained unaffected by the dilution. Again, the gold-doped SnO_2_ sensors using architecture A exhibited extremely low film resistances *R_a_* = 0.15 kΩ with low sensor response (*S* = 1.05 ± 0.01).

The sensor performance results are consistent with the materials characterization for sensor architecture A. In order for individual metal particles to bridge between the electrodes and thereby lower sensor resistances, the additives would have to be on the order of 10 μm. As seen in Table 1, XRD Scherrer analysis of the gold-doped SnO_2_ indicated an average gold crystallite size of 65 nm, and TEM imaging (Figure 4) confirms the XRD results. The individual Au particles are three orders of magnitude smaller than the electrode spacing and are not sufficiently large to bridge the electrodes. However, the TEM imaging indicated the Au particles are not uniformly dispersed in the SnO_2_. The regions of high Au concentrations can lead to locally very high conductivities near the electrodes, and thereby reduce the overall film resistance. The locally high Au concentrations and resulting increased rate of electron percolation would not be affected by diluting the dispersion with undoped SnO_2_. The low film resistances observed with both the 3.5 wt.% and the 0.35 wt.% Au loadings are consistent with this theory.

In terms of the bulk additive loadings in the films, the levels used in the current work are consistent with previous studies where the additives did lead to improved sensor performance. For example, gold loadings from 0.1–10 wt.% in tin dioxide enhanced sensor performance in the studies by Shimizu *et al.* (for target gas H_2_) [28], Sung *et al.* (for target gas CH_4_) [9], Ramgir *et al.* (for target gas CO) [13], Wang *et al.* (for target gas CO) [15], and Elmi *et al.* (for target gases C_6_H_6_, CO, and NO_x_) [4].

Regarding the sensor performance for sensor architecture B (colloidal Au-doped SnO_2_); this architecture exhibited a noticeable increase in the absolute resistance of the film *R_a_* consistent with other studies of Au-doped films compared to undoped films [7,12,29]. However, this sensor architecture yielded poor signal behavior with a low average sensor response of 2.2, and an average time response of 44 s. Another performance feature of this architecture is the non-ideal response behavior upon exposure to the target gas, *i.e.*, the non-stable gas response resistance *R_g_* and the inability for the resistance to return to the initial resistance Ra. This behavior, which was observed for multiple sensors with architecture B, may be caused by impurities originating from the colloidal dispersion, as suggested by Oh *et al.* [30].

### 3.3. Characterization of Sensor Performance: Localized Au Additives

**Figure 7.**
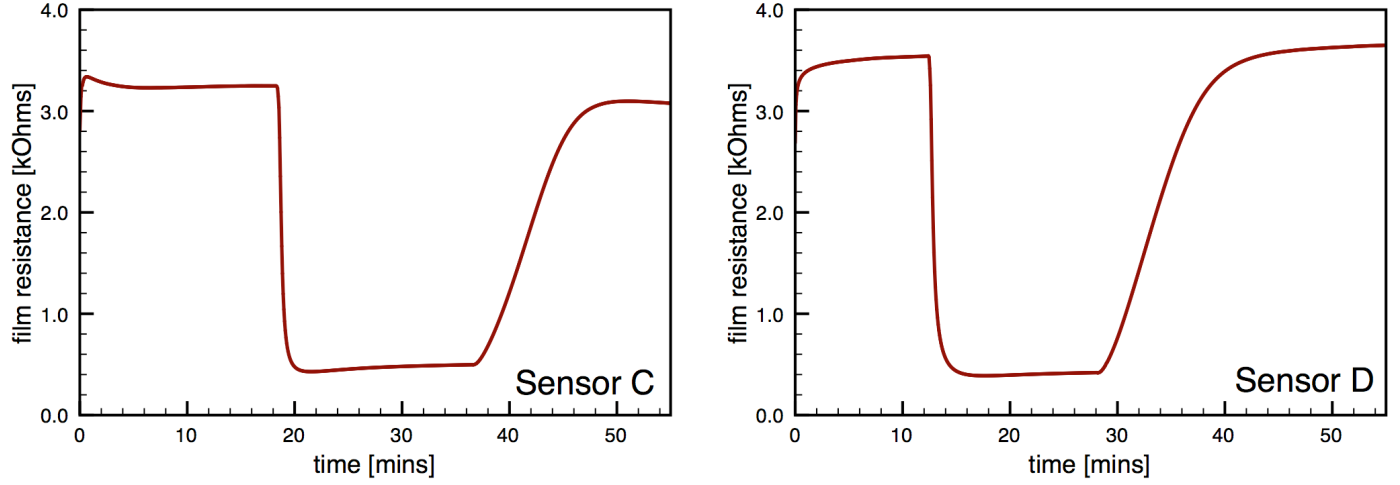
Sensor performance for localized gold additives; sensor architectures C and D.

Figure 7 presents typical sensor performance for the sensor architectures where the Au additives were localized to specific layers of the SnO_2_ film (sensor architectures C and D). For the ion sputtered Au (Sensor C), two layers of SnO_2_ dispersions were added before the sputtered Au layer was deposited. These results show noticeable improvement over previous architectures where the Au additives were distributed throughout the film. By avoiding locally high concentrations of additives near the electrodes this architecture produces high quality sensor signals with clear responses to the changes in the CO flow. The Au-sputtered sensors yielded an average sensor response of 6.1 and time response of 60 s.

To further consider controlling the location of the metal additives within the SnO_2_, a layered version of the CS materials was considered (Sensor D). Instead of sputtering the Au on the surface of the undoped SnO_2_ film, gold-doped SnO_2_ powders generated in the combustion facility were deposited to form the top layer of the sensor. The layered CS Au sensors yielded an average sensor response of 11.3 and time response of 51 s. This architecture provides an improvement of over 85% compared to the sputtered gold additive with an appreciable estimated sensitivity to 5 ppm CO based on partial pressure relationship [1].

### 3.4. Discussion of Sensor Performance Trends

An additional feature of the architecture with additives localized to the outer layers is the baseline resistance is comparable to the undoped SnO_2_ sensors [19]. As observed with the Sensor B architecture, there is an obvious increase in *R_a_* when additives are introduced to the sensing film. Since the additives for Sensors C and D are localized away from the electrodes, the absolute resistance remains nominally unaltered. In other words, although the additives and the local oxygen adsorbates may affect the conductivity due to the presence of metallic Au additives [11,28] in the top layer, the overall film resistance *R_a_* is dominated by the undoped SnO_2_ region. With the introduction of CO the dramatic decrease in resistance in the outermost layer due to the catalytic activity of the additives dictates the bulk resistance *R_g_* and contributes to the high sensing response. Other studies have demonstrated similar enhancement in performance with layered architectures [8,10,17,31,32,34].

**Figure 8.**
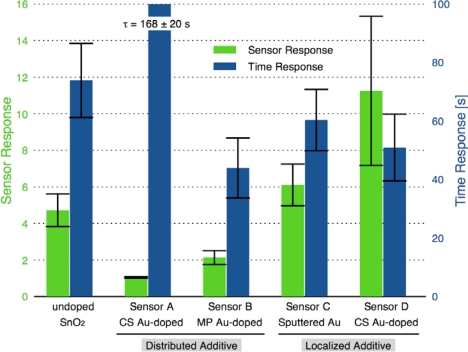
Comparison of sensor response and time response of the distributed and localized Au additive architectures for sensor operating temperature of 330 °C.

**Figure 9.**
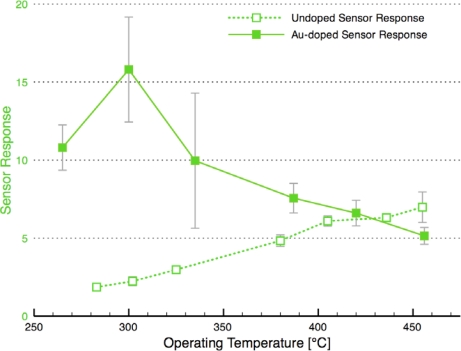
Comparison of the temperature dependence of Au-doped (Sensor D architecture) and undoped tin dioxide sensors.

Figure 8 compares the sensor response and time response of each of the sensor architectures. There is dramatic improvement in the sensor performance for the CS layered sensor compared to the other Au-doped and undoped SnO_2_ materials. A comparison of the temperature dependence of the undoped and CS layered Au-doped sensor further supports the improvement in the sensor characteristics, as presented in Figure 9. The sensor response and time response are improved and the optimum sensor operating temperature has been lowered (from over 450 °C for the undoped sensor to ∼300 °C for the Au-doped sensor). Similar shifts in the temperature dependence have been observed with Au-doped systems [7,12,13,15,16]. However, other studies suggest higher peak operating temperatures with Au-doped systems and CO gas sensing [4,8]. The discrepancies may be explained by differences in the additive incorporation and size distribution [33].

The underlying sensing mechanism that can explain the observed results between the distributed and the localized gold architectures is challenging to identify without more extensive material and surface chemical characterization. In fact, Korotcenkov and coworkers state that the mechanism of the influence of surface modification is ‘obscure’ [17]. Metal additive studies suggest a number of important sensing and transduction determinants even though not all explanations are reconcilable [17,28,34]. It is well known that noble metal-SnO_2_ powders show improved catalytic conversion of CO to CO_2_ beyond 200 °C ([17] and refs there in). As a result, according to Sahm *et al.* [34] the addition of a localized additive layer should result in a decrease in sensor response because the top layer will preferentially oxidize CO with minimal change to the oxygen adsorbate concentration of the undoped SnO_2_ film beneath. This is opposite to the observations made in the current study. Shimizu *et al.* [28] and Korotcenkov *et al.* [17] explain performance improvement with Au additives via the alteration of oxygen adsorption kinetics. Specifically, the reduction in oxygen re-adsorption rates during CO exposure resulting in enhanced sensitivity to the target gas. This hypothesis cannot be confirmed for the films studied in this work, without quantitative evaluation of the surface sites for the different film architectures.

**Figure 10.**
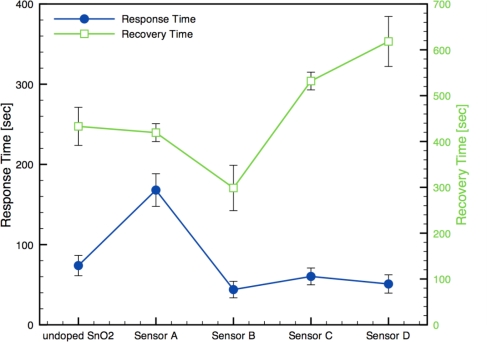
Comparison of the response and recovery times for the sensor architectures.

Alternatively, the physical film architecture (*i.e.*, pore size and structure) can influence the diffusion of gases through the films and affect the response and recovery times. Figure 10 presents response and recovery times for each sensor architecture, including the undoped-SnO_2_ sensor. The results show both response and recovery times are comparable in magnitudes to other similar studies (1–10 min) [8,12,13,14,16]. The response time results present a clear improvement when Au additives are incorporated except for the Sensor A architecture where the shunting effect was observed. The recovery times demonstrated noticeable increase with the localized sensor architectures. In other words, the improved sensor response and time response are associated with longer recovery times.

## 4. Conclusions

The combustion-generated Au-doped SnO_2_ materials yielded sensors with excellent sensor response and time response, as well as optimum operating temperatures that are reasonably low for reduced power consumption. The results of this study also demonstrate the importance of additive location in SnO_2_ gas sensors at multiple scales; including additive distribution at the μm level within the sensing film and at the nm level in the SnO_2_ matrix. Additive distribution is a multi-dimensional design criterion that can be used to further optimize sensor performance. At the same time, characterizing the integration of additives is critical to deconvolve the potential effects of additive distribution over, for example, chemical attributes when distribution is not a design parameter. Quantifying sensor additive architecture is an important and necessary metric for comparing sensor performance and understanding the fundamental mechanisms important in doped metal oxide sensors.

## Figures and Tables

**Table 1.** Summary of Au/SnO_2_ sensor architectures considered. All SnO_2_ materials were generated by combustion synthesis with an average SnO_2_ particle size of 15 nm based on XRD analysis, while Au additive synthesis and properties are indicated respective to the architectures.

**Au synthesis method**	**Bulk Au loading/Au particle size**	**Au distribution/Film thickness**	**Schematic of sensor architecture**
**Sensor Architecture: A**			

Combustion	3.5 wt% (or 0.35 wt%)^a^/65 nm^b^	*Distributed* throughout the SnO_2_ film/10 μm	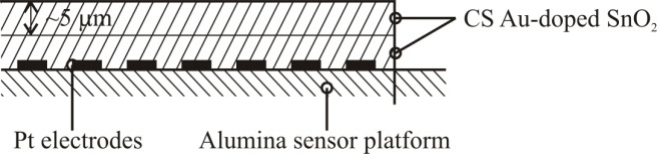
**Sensor Architecture: B**			

Metal Precipitation	0.2 wt%^c^/15 nm^d^	*Distributed* throughout the SnO_2_ film/10 μm	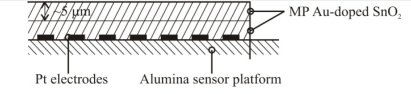
**Sensor Architecture: C**			

Ion Sputtering	5 wt%^e^/32 nm^b^	*Localized* to the outermost layer of film/10 μm + sputtered layer of Au nanoparticles	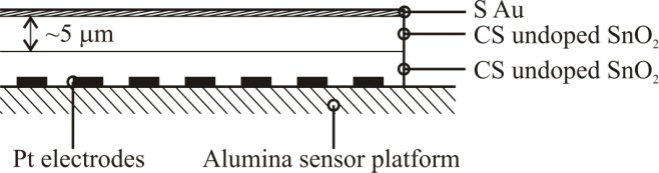
**Sensor Architecture: D**			

Combustion	3.5 wt%^a^ for the outermost layer/65 nm^b^	*Localized* throughout the outermost layer of SnO_2_/10 μm SnO_2_ + 5 μm CS Au-doped SnO_2_	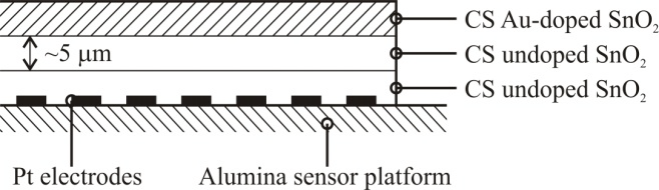

a Based on EMPA.

b Based on XRD Scherrer analysis.

c Estimate, see text for details.

d Based on TEM image analysis.

e Based on XEDS.
